# 749 Healthcare Disparity Increases Risk of Dog Bites in the Pediatric Population

**DOI:** 10.1093/jbcr/irac012.302

**Published:** 2022-03-23

**Authors:** Christopher Carballo, Justin Faulkner, Austin Gratton, Samuel Baughman, Elizabeth Acquista, William Powers, James R Yon

**Affiliations:** New Hanover Regional Medical Center, Wilmington, North Carolina; New Hanover Regional Medical Center, Wilmington, North Carolina; Novant Health/New Hanover Regional Medical Center, Wilmington, North Carolina; New Hanover Regional Medical Center, Wilmington, North Carolina; New Hanover Regional Medical Center, Wilmington, North Carolina; New Hanover Regional Medical Center, Wilmington, North Carolina; New Hanover Regional Medical Center, Wilmington, North Carolina; Novant Health/New Hanover Regional Medical Center, Wilmington, North Carolina

## Abstract

**Introduction:**

Long known to be a widespread source of injury, dog bites continue to occur and can frequently be devastating. There is lasting economic and personal hardship associated with this as many attacks can be severe, especially in children. This study describes the differences between dog bites and all other bites in the trauma patient population.

**Methods:**

We included all patients from the National Trauma Data Bank (NTDB) from 2007 to 2016. All patients with an E-code for any type of bite were included. The following predictors were examined: year, age, gender, race, ethnicity, transfer, injury type, ED disposition, and ISS ≥15. Standard t-test for age, chi-squared test for all categorical variables. Wilcoxon test for non-normal quantitative variables. Statistical analyses were performed using R version 4.0.2 statistical software. “Under resourced” will be defined as Self-Pay and Medicaid. “Not known” is removed and replaced with NAs. All other insurance types are included in “Normal”.

**Results:**

All variables tested as significant. All variables analyzed tested as significantly different between dog bites and other animal bites. When the group, pediatric subset, and adult subset are evaluated (Tables 1), pediatric patients are more likely to be dog bite patients if they are from an under resourced background (OR 1.3, p-value < 0.001). Distribution of bites are not significantly different from Low to Normal resource adult patients.

**Conclusions:**

Dog bites continue to be a public health problem and are more likely to be severe or fatal than bites from other animals and humans. Better methods of injury prevention and education need to be established. Children from under resourced backgrounds are at an increased risk of suffering a dog bite and all physicians should screen for and educate patients and families about this risk.

